# Trans*forming the Life Course: New Directions for Critical Gerontology

**DOI:** 10.1093/geroni/igaf122.3678

**Published:** 2025-12-31

**Authors:** Jen Justice

**Affiliations:** Miami University, Oxford, Ohio, United States

## Abstract

The life course perspective proposes life timelines to which individuals must adhere in order to minimize disruptions to their developmental trajectory and age optimally. These normative timelines are constructed and enforced with the ultimate goal of upholding traditional Western values such as reproductive futurity and capitalistic productivity. The framework is predicated on the assumption of a homogenous society; equal opportunity and desire to access each life transition is assumed and thus deviation in their sequence or timing is seen as a personal failure rather than cultural difference. The life course perspective introduces normative constructions of time that lack applicability to individuals of non-normative identities, whose lives often do not follow the linear and teleological progression proposed by the model, instead unfolding in nonlinear, nonconsecutive, and multidirectional paths. By delineating ‘on-time’ and ‘off-time’ events and, by extension, ‘right’ and ‘wrong’ ways to age, the life course perspective acts as a mechanism of oppression employed by social institutions to pathologize individuals of non-normative identities, particularly in later life. Drawing from critical, cultural, queer, and trans* studies and bringing these disciplines into dialogue with gerontological theory, this paper offers a theoretical trans*formation of time, aging, and the life course.

